# High production of pro-inflammatory cytokines by maternal blood mononuclear cells is associated with reduced maternal malaria but increased cord blood infection

**DOI:** 10.1186/s12936-018-2317-2

**Published:** 2018-05-10

**Authors:** Carlota Dobaño, Tamara Berthoud, Maria Nelia Manaca, Augusto Nhabomba, Caterina Guinovart, Ruth Aguilar, Arnoldo Barbosa, Penny Groves, Mauricio H. Rodríguez, Alfons Jimenez, Lazaro M. Quimice, John J. Aponte, Jaume Ordi, Denise L. Doolan, Alfredo Mayor, Pedro L. Alonso

**Affiliations:** 1 ISGlobal, Hospital Clínic-Universitat de Barcelona, Carrer Rosselló 153 (CEK Building), 08036 Barcelona, Catalonia Spain; 2 Centro de Investigação em Saúde de Manhiça (CISM), Maputo, Mozambique; 3 CIBER Epidemiología y Salud Pública (CIBERESP), Barcelona, Spain; 4 Queensland Institute of Medical Research, Brisbane, Australia

**Keywords:** Malaria, *P. falciparum*, Pregnancy, Cytokines, Immunity, Pathology, Cord

## Abstract

**Background:**

Increased susceptibility to malaria during pregnancy is not completely understood. Cellular immune responses mediate both pathology and immunity but the effector responses involved in these processes have not been fully characterized. Maternal and fetal cytokine and chemokine responses to malaria at delivery, and their association with pregnancy and childhood outcomes, were investigated in 174 samples from a mother and child cohort from Mozambique. Peripheral and cord mononuclear cells were stimulated with *Plasmodium falciparum* lysate and secretion of IL-12p70, IFN-γ, IL-2, IL-10, IL-8, IL-6, IL-4, IL-5, IL-1β, TNF, TNF-β was quantified in culture supernatants by multiplex flow cytometry while cellular mRNA expression of IFN-γ, TNF, IL-2, IL-4, IL-6, IL-10 and IL-13 was measured by quantitative PCR.

**Results:**

Higher concentrations of IL-6 and IL-1β were associated with a reduced risk of *P. falciparum* infection in pregnant women (p < 0.049). Pro-inflammatory cytokines IL-6, IL-1β and TNF strongly correlated among themselves (ρ > 0.5, p < 0.001). Higher production of IL-1β was significantly associated with congenital malaria (p < 0.046) and excessive TNF was associated with peripheral infection and placental lesions (p < 0.044).

**Conclusions:**

Complex network of immuno-pathological cytokine mechanisms in the placental and utero environments showed a potential trade-off between positive and negative effects on mother and newborn susceptibility to infection.

**Electronic supplementary material:**

The online version of this article (10.1186/s12936-018-2317-2) contains supplementary material, which is available to authorized users.

## Background

In malaria endemic areas, the negative outcomes of *Plasmodium falciparum* infections on health concentrate in infants and pregnant women [1]. Parasites accumulate in the placenta during pregnancy, and this has been associated with adverse consequences in mother and fetus [2]. Cellular responses have been involved in pathology and immunity to malaria, but cytokines mediating these processes have not been fully characterized [3–5]. Lymphoproliferative responses and T_H_1 cytokines like IL-12, IFN-γ and IL-2 are generally depressed during gestation, particularly in primigravidae [6–8], in response to malarial and non-malarial antigens and mitogens. Alterations during pregnancy appear to be due to modulation of cellular immunity rather than involve a malaria-specific phenomenon [9].

Systemic cytokine profiles during pregnancy are biased towards T_H_2, a pre-requisite for successful gestations [10], with predominance of IL-4 and IL-5. Overall, *P. falciparum* induces placental immune responses involving T_H_1 and T_H_2 pathways [11] but increasing T_H_1/pro-inflammatory responses involving IL-1β, IL-6, IL-8 and TNF to a greater extent. However, the secretion of the anti-inflammatory cytokine IL-10 is also elevated presumably to control the negative effects of excessive inflammation [12, 13]. Production of IFN-γ by placental cells (natural killer and CD4^+^ T_H_1 cells) has been associated with protection against placental malaria [14–17].

Placental malaria influences fetal immune responses [18], and may affect infant health [19]. Prenatal immune priming to *Plasmodium* is common in endemic areas [20–24]. Maternal infection may affect the T_H_1/T_H_2 balance in cord through IL-10 production by T regulatory cells (Tregs), which is elevated in parasitaemic women [25], being a mechanism of neonatal immune suppression to reduce T_H_1 responses [26–29]. Cells of neonates born to infected mothers have altered IFN-γ and TNF responses after stimulation with toll-like receptor ligands [30, 31]. Treg frequencies are higher in cord blood from neonates born to mothers infected early in pregnancy, and activated CD4^+^ T cells and myeloid dendritic cells are more common in neonates born to mothers with active placental infection at the time of delivery [32]. How these abnormalities impact acquisition of immunity in infancy is not clear, however recent studies show that increased inflammatory mediators in cord blood due to prenatal exposures are inversely associated with risk of severe malaria in early life [33, 34].

In this study, the factors that affect maternal and fetal cytokine responses to *P. falciparum* antigens at delivery were analysed. The investigation focused on three groups of cytokines including (i) those that are important for the control of malaria infection, i.e. T_H_1 and pro-inflammatory responses, (ii) those that are important to restrict the pathology associated with exacerbated inflammation, i.e. regulatory cytokines, and (iii) those that are important to ensure a successful pregnancy, i.e. T_H_2 responses. To this end, a multiplex panel of cytokines that included a sufficient breadth of cytokine functions that was successfully applied in previous studies by the same group was used. The analysis was completed with an assessment of the association of those cytokines with pregnancy and childhood outcomes. The study included maternal and cord samples collected from a mother–child cohort in Mozambique, in the context of a randomized, double-blind, placebo-controlled trial (AgeMal, ClinicalTrials.gov: NCT00231452) [35].

## Methods

### Study area

The study was conducted at the Centro de Investigação em Saúde de Manhiça (CISM) in southern Mozambique [36]. CISM runs demographic and morbidity surveillance systems at the Manhiça District Hospital (MDH) and nearby health posts where standardized information on paediatric outpatient visits and admissions is collected. Recruitment and follow up of participants were done at the Maragra Health Post (MHP) (September 2005–March 2009). Transmission of *P. falciparum* is perennial with marked seasonality (warm rainy season November–April, cool dry season rest of year) and of moderate intensity.

### Study design

HIV-negative pregnant women resident in Manhiça were recruited during the third trimester of pregnancy at the antenatal clinic [35]. Exclusion criteria included birth weight < 2 kg, same gender twins, congenital malformations, birth asphyxia or apparent health problems. 349 eligible newborns were enrolled.

Samples were collected at delivery: 10 mL of maternal venous blood (EDTA vacutainer), 8 mL of cord blood (EDTA vacutainer), blood slides, bloodspots onto filter papers and placental tissue. When delivery occurred outside the maternity, only maternal blood samples were collected.

Children were followed up until age 24 months. Weekly active case detection was conducted from birth to age 10.5 months, and monthly home visits from 10.5 to 24 months. Children with fever (axillary temperature ≥ 37.5 °C) or history of fever in the preceding 24 h were taken to the MHP for examination, parasitaemia and haematocrit assessment. Passive case detection was carried out at the MHP and MDH to monitor attendances to outpatient/inpatient clinics.

### Laboratory procedures

Parasitaemia was quantified in blood slides at CISM following standard quality-controlled procedures [35]. Haematocrit was measured after centrifugation in heparinized microcapillary tubes with a haematocrit reader (Hawksley & Sons Ltd, Lancing, UK), and full blood counts performed using a KX-21N cell counter (Sysmex Corporation, Kobe, Japan). Placental biopsies collected from the maternal side were placed into 10% neutral buffered formalin and presence of parasites or pigment evaluated [37]. Real-time quantitative PCR (qPCR) for *P. falciparum* 18SrRNA (ABI PRISM 7900HT Fast Real-Time System) using TaqMan^®^ probe and FAM/TAMRA, in duplicates, in filter papers of maternal and cord blood [38].

### Cell stimulation and cytokine and chemokine quantification

Peripheral blood mononuclear cells (PBMC) and cord blood mononuclear cells (CBMC) were isolated using a Ficoll-Hypaque 1.077 gradient, resuspended in complete medium (RPMI 1640 culture medium with glutamine and antibiotics plus decomplemented 10% fetal calf serum), and stimulated as previously reported [39]. Briefly, 1.2 million fresh PBMC and CBMC were incubated with 20 μL of a 3D7 *P. falciparum* schizont extract corresponding to 2 million synchronized infected red blood cells (iRBC, lysed by freezing–thawing cycles, endotoxin and Mycoplasma tested), or with 20 μL of lysate from uninfected RBC (uRBC, control) in 24-well plates in a total volume of 590 μL. Supernatants were collected after 24, 48 or 72 h cultures and frozen at − 80 °C. The PBMC and CBMC pellets were collected in Trizol (InVitrogen) and frozen at − 80 °C. Supernatants were shipped in dry ice to ISGlobal where they were thawed and IL-12p70, IFN-γ, IL-2, IL-10, IL-8, IL-6, IL-4, IL-5, IL-1β, TNF, TNF-β quantified with the Bender Human T_H_1/T_H_2 11plex FlowCytomix Multiplex Kit (MedSystems, Aachen, Germany) as previously described [39]. Cytokine production in culture supernatants of unstimulated maternal and neonatal samples (uRBC) was not subtracted from the iRBC stimulated samples [40], but shown side by side as it is possibly biologically relevant [41–43]. Limits of detection for cytokine/chemokine concentrations are as reported [39].

Messenger RNA (mRNA) and complementary DNA (cDNA) from 44 maternal PBMC samples stimulated for 24 h with parasite iRBC lysate and uRBC control were purified and cytokine transcript levels measured at QIMR [44]. RNA was extracted from PBMC in Trizol (Invitrogen, Carlsbad, CA, USA). Total RNA was reverse transcribed using Superscript™III R (Invitrogen). cDNA was quantified by reverse transcriptase qPCR (RT-qPCR) in triplicate using commercially available primers and probes (Taqman™, Applied Biosystems, CA, USA) specific for human IFN-γ (Hs00174143_m1), TNF (Hs00174128_m1), IL-2 (Hs00174114_m1), IL-4 (Hs00174122_m1), IL-6 (Hs00174131_m1), IL-10 (Hs00174086_m1), IL-13 (Hs00174379_m1) and ribosomal protein L13a (RPL13a) (Hs03043885_g1) as reference gene (Apte JI 2010, unpublished). Thermal cycler parameters (Corbett Rotor-Gene 3000, Mortlake, Australia) were 95 °C for 2 min and 60 °C for 30 s, 45 cycles at 95 °C for 5 s, and 60 °C for 30 s. Results were calculated by the number of molecules of each sample as determined from the standard curve for each gene, which was then standardized against the number of molecules of RPL13a (reference gene) for that sample.

### Definitions and statistical methods

Pregnant women were classified into primigravidae (PG) and those with at least one previous pregnancy (multigravidae, MG). Maternal age was categorized as ≤ 20, 21–24 and ≥ 25 years. Distance to the river was calculated with Hawth’s tools in ArcGIS software (ESRI) as the mean distance of each neighbourhood centroid to the Incomati river, and neighbourhoods classified as those adjacent to the river (mean distance ≤ 2.5 km) or those at a greater distance (> 2.5 km) [45]. Season during pregnancy was defined as dry if the gestation period included May–October, and rainy if it included ≥ 4 rainy months (November–April). Maternal malaria was defined as parasites in peripheral blood by microscopy and/or qPCR. Placental malaria was defined as parasites and/or pigment by histology. Placental inflammation was defined as presence of > 5 mononuclear cells and/or polymorphonuclear leukocytes in intervillous spaces, assessed in 10 high power fields at 400× [2]. Congenital malaria was defined as parasites on cord blood by qPCR. The primary case definition of clinical malaria in children was fever or history of fever plus *P. falciparum* parasites of any density [35].

Cytokine and chemokine concentrations (pg/mL) were logarithmically transformed. Continuous variables were compared by ANOVA and categorical variables by Fisher’s exact test. Spearman’s test and scatter plots assessed correlations among cytokines and chemokines. The Bonferroni correction was applied to p values to adjust for multiple comparisons in correlation analyses. Linear regression models were used to assess whether age, parity, neighbourhood, season (rainy *vs* dry), use of insecticide-treated net (ITN), or use of indoor residual spraying (IRS), affected the magnitude of maternal and fetal cytokine and chemokine responses and whether there was an interaction between parity and these variables. Analyses were done univariate and multivariable, adjusting for all the other variables. When a significant interaction with parity was found, an additional set of regression analyses was performed stratifying women by parity. Logistic (for infection and inflammation) or linear (for weight and haematocrit) regression models were used to evaluate the effect of doubling the levels of maternal and fetal cytokines and chemokines on pregnancy outcomes: *P. falciparum* infection (maternal, placental, cord), placental inflammation, maternal haematocrit and birth weight. Analyses were adjusted for age, parity, season, neighbourhood, ITN, IRS and, when applicable, *P. falciparum* infection. In women with peripheral parasitaemia, Spearman’s correlations between parasite density (microscopy and qPCR) and cytokine and chemokine levels, and between parasite density and placental inflammation, were performed. Adjusted negative binomial regression models evaluated the effect of doubling the levels of maternal and fetal cytokines and chemokines on the incidence of multiple malaria episodes in children up to 12 months of age. Significance was defined at p < 0.05. Crude p values are interpreted for internal coherence, consistency of results and biological plausibility. Analyses were performed using Stata/SE 10.1 (College Station, TX, USA).

## Results

### Kinetics of cytokine and chemokine production by cells after *Plasmodium falciparum* antigen stimulation

To determine the optimal timepoint for cytokine and chemokine secretion in supernatants after stimulation with iRBC and uRBC lysates, a pilot study was conducted with 44 maternal PBMC and 44 matched CBMC samples (28 aparasitaemic and 16 parasitaemic mothers). Cytokine and chemokine concentrations were compared at 24, 48 and 72 h (n = 264 supernatants). In general, there was not a significant difference in cytokine and chemokine concentrations between timepoints. For some cytokines (IFN-γ, IL-6, IL-10, TNF) responses tended to be higher at 24 h and thus this timepoint was chosen for subsequent studies. Another pilot study was conducted to determine the optimal timepoint for cytokine mRNA production with 11 maternal PBMC samples and the 24 h incubation was also selected as the most appropriate.

### Correlations among cytokine and chemokine proteins and mRNAs

**Fig. 1:**
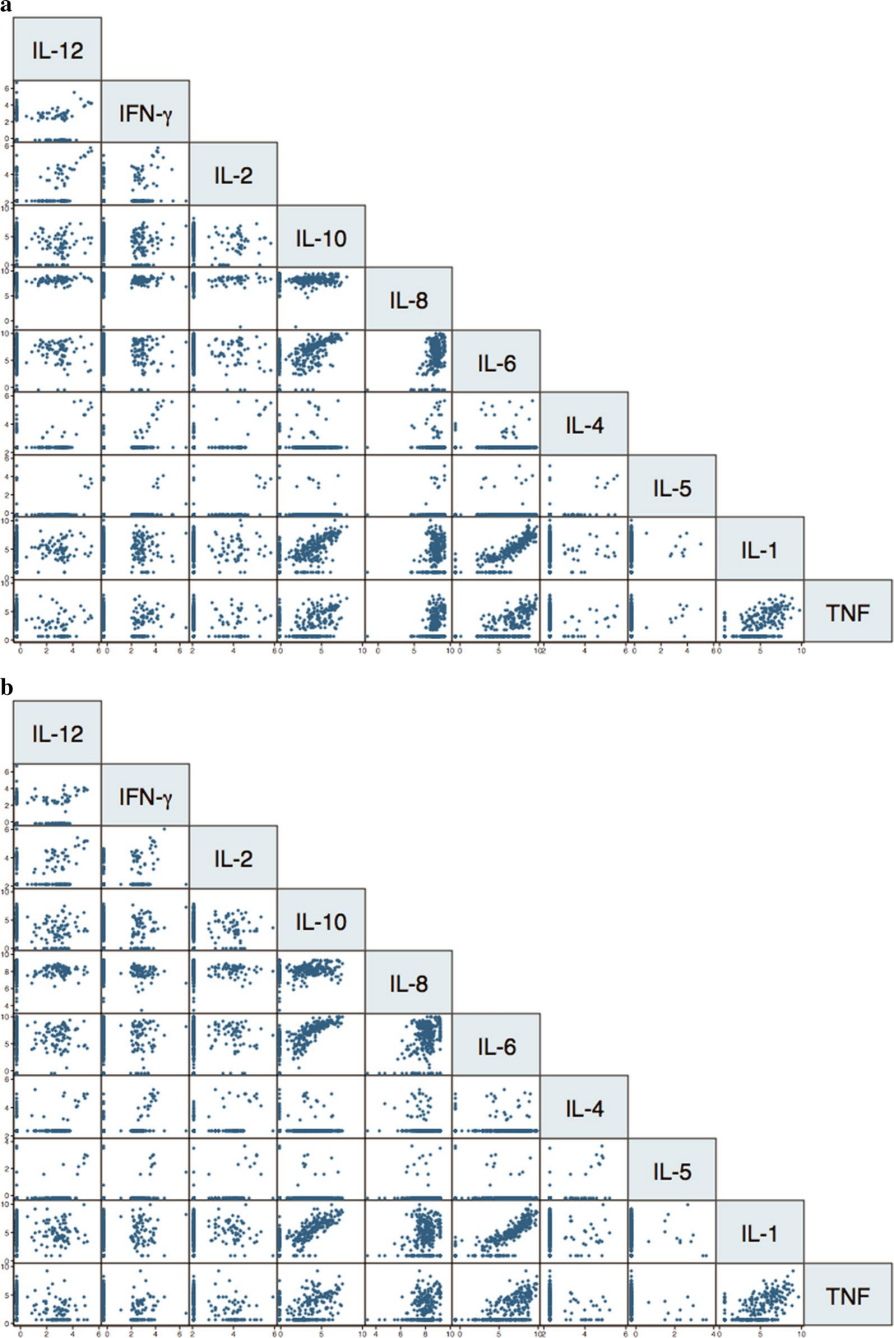
Correlations among cytokines and chemokines secreted by peripheral blood mononuclear cells in culture supernatants. Stimulation with a lysate of *Plasmodium falciparum* infected erythrocytes (**a**) and a lysate of uninfected erythrocytes (**b**). Significant Spearman rho (ρ) coefficients and p values (Bonferroni corrected) are reported in Table 2

Analyses reported here are based on cytokine and chemokine data obtained from 174 women (Table 1, see Additional file 1) in whom PBMC and/or CBMC of enough quantity and quality were obtained, cells could be stimulated with antigen ex vivo and supernatants processed. Table 2 presents the Spearman’s ρ coefficients and p values (Bonferroni adjusted) of the correlation analyses. Pro-inflammatory cytokines and chemokines IL-1β, IL-6, TNF and IL-8 were highly correlated (Fig. 1 PBMC supernatants, see Additional file 2 CBMC supernatants, Additional file 3 mRNA). A significant correlation was also found between pro-inflammatory cytokines and the regulatory cytokine IL-10. Moderate correlations were seen among T_H_1 cytokines, T_H_2 cytokines, and also between T_H_2 and T_H_1 cytokines (Table 2). Inverse correlations were observed for IL-8 and IFN-γ or IL-12. No substantial differences were found between peripheral and cord cytokines but peripheral responses tended to be slightly higher (see Additional file 4). Some cytokine and chemokine responses appeared not to be malaria-specific, as spontaneous release of pro-inflammatory cytokines was also found in control stimulations (see Additional file 5).

**Table 1:** Parasitological, demographic and epidemiological characteristics of the women whose peripheral and cord blood samples were assessed for cytokine and chemokine secretion following cell stimulation with *Plasmodium falciparum* lysate

	Peripheral blood samples (n = 172)	Cord blood samples (n = 174)
Placental infection	35 (20.3)^a^	35 (20.1)
Peripheral infection	31 (18)	30 (17.2)
Cord infection	6 (3.5)	6 (3.4)
Placental inflammation	9 (5.2)	9 (5.2)
Age		
15–20	60 (34.8)	61 (35.5)
20–25	48 (27.9)	48 (27.6)
> 25	64 (37.2)	65 (37.8)
Parity		
Primigravidae	48 (27.9)	47 (27)
Neighbourhood		
1	75 (43.6)	76 (43.7)
2	97 (56.4)	98 (56.3)
ITN use	21 (12.2)	20 (11.5)
IRS use	81 (47.1)	86 (49.4)
Season		
Dry	77 (44.8)	77 (44.2)
Child group		
Control	61 (35.4)	58 (33.3)
Late exposure	51 (29.6)	55 (31.6)
Early exposure	60 (34.8)	61 (35.0)

Numbers and percentages (in parenthesis) are shown

Parasite densities (geometric mean, 95% CI) in infected women were: peripheral microscopy (n = 13) 41,364, 1585–81,144; peripheral qPCR (n = 29) 0.98, 0.28–3.43; placental parasitaemia 5.76, 0.73–45.42; cord qPCR 0.05, 0.01–0.15

The combination between peripheral malaria vs placental malaria vs congenital malaria vs placental inflammation is shown in Additional file 1

*ITN* insecticide-treated net, *IRS* indoor residual spraying

^a^By histology: 3 acute infections, 1 chronic infection, 31 past infections

**Table 2:** Correlations among cytokines and chemokines in supernatants

Cytokines and chemokines	Spearman’s rho [ρ] coefficient	p value (Bonferroni adjusted)
Pro-inflammatory		
Peripheral blood		
IL-1β and IL-6	0.9	< 0.001
IL-6 and TNF	0.6	< 0.001
TNF and IL-1β	0.5	< 0.001
IL-6 and IL-8	0.3	< 0.024
Cord blood		
IL-1β and IL-6	0.9	< 0.001
IL-6 and TNF	0.6	< 0.001
TNF and IL-1β	0.5	< 0.001
Pro-inflammatory and regulatory		
Peripheral blood		
IL-6 and IL-10	0.6	< 0.001
IL-1β and IL-10	0.6	< 0.001
TNF and IL-10	0.4	< 0.001
Cord blood		
Il-6 and IL-10	0.6	< 0.001
IL-1β and IL-10	0.6	< 0.001
TNF and IL-10	0.4	< 0 .001
IL-2 and IL-10	0.3	< 0.015
T_H_1		
Peripheral blood		
IL-12 and IL-2	0.3	0.018
IL-2 and IFN-γ	0.3	0.004
IL-12 and IFN-γ	0.3	0.110
Cord blood		
IL-12 and IL-2	0.3	0.018
IL-2 and IFN-γ	0.3	0.004
IL-12 and IFN-γ	0.3	0.110
T_H_2		
Cord blood		
IL-4 and IL-5	0.4	< 0.001
T_H_1 and T_H_2		
Peripheral blood		
IL-4 and IFN-γ	0.3	0.001
Cord blood		
IL-4 and IFN-γ	0.5	< 0.001
IL-4 and IL-12	0.3	< 0.001
IL-4 and IL-2	0.3	< 0.001
T_H_1 and chemokines		
Peripheral blood		
IL-8 and IFN-γ	− 0.3	< 0.001
Cord blood		
IL-8 and IL-12	− 0.3	0.011

The table shows results that are statistically significant or that are close to significance, after adjustment for multiple comparisons

**Fig. 2:**
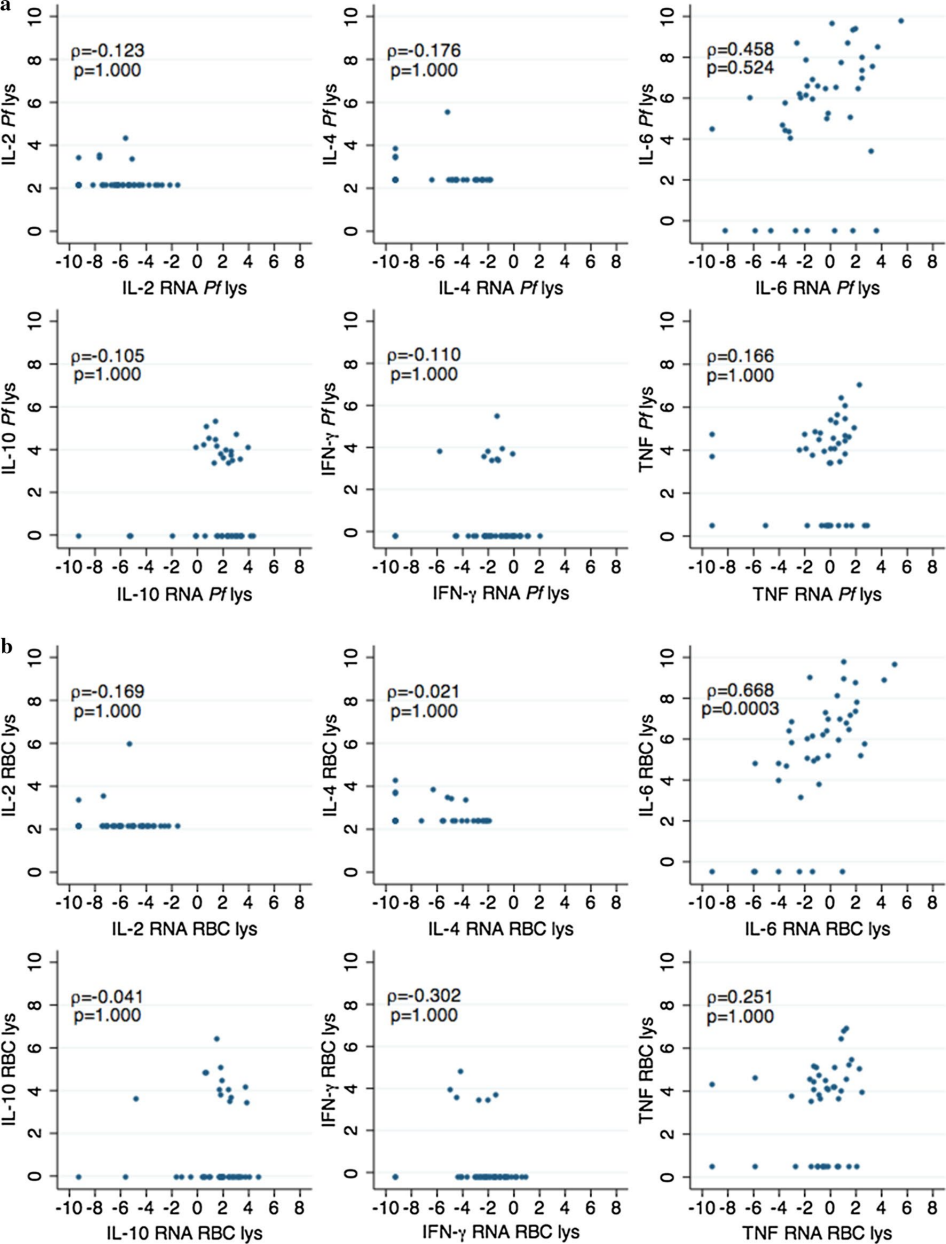
Correlations between cytokines produced by peripheral blood mononuclear cells in culture supernatants and mRNA within cells. Stimulation with a lysate of *Plasmodium falciparum* infected erythrocytes (**a**) and a lysate of uninfected erythrocytes (**b**), showing the rho (ρ) coefficients and p values (Bonferroni corrected). X axis: due to the data pre-processing of the cytokine RT-qPCR data (see “Methods”), in cases of very low responses negative values were obtained that are considered as zero

The correlations between secreted proteins and intracellular mRNA cytokines were also examined. For the positive IFN-γ, TNF, IL-10 and IL-6 responses, there was a high correlation between the levels of cytokines secreted in the supernatants and the mRNA levels contained within the PBMC (Fig. 2). However, a number of supernatant cytokine responses were below the detection limit of the FlowCytomix kit but were measurable as mRNA by RT-qPCR, and this rendered the correlations non-significant (Fig. 2). This could be because (i) the RT-qPCR method was more sensitive, and/or (ii) the mRNA transcribed in the cytoplasm was not translated into protein and secreted to the supernatant, and/or (iii) the cytokines/chemokines secreted were bound to receptors and internalized by the cells. For IL-2 and IL-4, concentrations were overall low. Subsequent analyses included only supernatant proteins due to the limited number of samples in which mRNA cytokine levels were quantified.

### Factors affecting secretion of cytokines and chemokines

Concentrations were significantly affected by age, parity, seasonality and ITN use, but not by neighbourhood or IRS. After ex vivo stimulation with iRBC and uRBC, PBMC from older women secreted significantly higher levels of IL-1β and IL-6 than younger women and the differences remained significant after adjustment (Table 3). Upon iRBC stimulation, CBMC from MG secreted significantly higher levels of IL-8 than PG women (Table 3). A significant interaction between age and parity was observed for IL-5 production by PBMC (p = 0.026). After stratifying the analyses by parity, higher IL-5 concentrations in PG but not in MG women were significantly associated with older age (Table 3).

**Table 3:** Factors affecting the concentration of cytokines and chemokines secreted in culture supernatants of peripheral and cord blood mononuclear cells after incubation with *P. falciparum*-infected erythrocyte (iRBC) or uninfected erythrocyte (uRBC) lysates

	Peripheral blood				Cord blood			
	Univariate		Multivariable		Univariate		Multivariable	
	Effect (95% CI)	p	Effect (95% CI)	p	Effect (95% CI)	p	Effect (95% CI)	p
Older age^a^								
IL-1β								
iRBC	3.44 (1.58;7.50)	0.017	4.42 (1.40;13.91)	0.025	NS		NS	
uRBC	3.33 (1.46;7.60)	0.008	NS		NS		NS	
IL-6								
iRBC	4.42 (1.79;10.91)	0.005	7.75 (2.02;29.8)	0.013	NS		NS	
uRBC	3.10 (1.18;8.15)	0.036	5.49 (1.31;22.9)	0.046	NS		NS	
IL-5^d^								
iRBC	37.7 (11.7;121.1)	< 0.001	38.3 (12.0;122.0)	< 0.001	NS		NS	
uRBC	NS		NS		NS		NS	
Parity^b^								
IL-8								
iRBC	NS		NS		NS		1.29 (0.65;2.55)	0.048
uRBC	NS		NS		NS		NS	
Dry season^c^								
IL-1β								
iRBC	0.25 (0.13;0.48)	< 0.001	0.23 (0.12;0.43)	< 0.001	0.23 (0.12;0.46)	< 0.001	0.21 (0.11;0.43)	< 0.001
uRBC	0.23 (0.12;0.47)	< 0.001	0.21 (0.11;0.42)	< 0.001	0.21 (0.10;0.42)	< 0.001	0.18 (0.09;0.37)	< 0.001
IL-6								
iRBC	0.25 (0.12;0.54)	< 0.001	0.25 (0.12;0.52)	< 0.001	0.14 (0.06;0.33)	< 0.001	0.14 (0.06;0.32)	< 0.001
uRBC	0.19 (0.08;0.41)	< 0.001	0.19 (0.09;0.42)	< 0.001	0.12 (0.05;0.27)	< 0.001	0.11 (0.05;0.26)	< 0.001
TNF								
iRBC	0.51 (0.27;0.95)	0.036	0.53 (0.28;1.00)	0.051	0.26 (0.14;0.49)	< 0.001	0.28 (0.15;0.53)	< 0.001
uRBC	0.40 (0.21;0.75)	0.005	0.40 (0.21;0.74)	0.004	0.32 (0.17;0.61)	0.001	0.34 (0.18;0.66)	0.002
IL-8								
iRBC	0.66 (0.48;0.91)	0.012	0.65 (0.47;0.90)	0.011	NS		NS	
uRBC	0.67 (0.52;0.86)	0.002	0.68 (0.52;0.88)	0.003	0.70 (0.51;0.96)	0.026	0.67 (0.48;0.93)	0.017
IL-2								
iRBC	NS		NS		0.75 (0.57;0.99)	0.046	0.74 (0.57; 0.96)	0.027
uRBC	NS		NS		NS		NS	
IL-4								
iRBC	NS		NS		0.79 (0.63;0.98)	0.033	0.79 (0.63;1.00)	0.047
uRBC	NS		NS		NS		NS	
Insecticide treated net use								
IL-10^e^								
iRBC	NS		NS		3.00 (1.06;8.51)	0.040	4.17 (1.34;12.98)	0.015
uRBC	NS		NS		3.25 (1.16;9.13)	0.026	3.38 (1.20;12.20)	0.025

The following variables were included in the multivariable regression analyses: peripheral and placental infection, placental inflammation, age, parity, neighbourhood, season, use of insecticide treated net and use of indoor residual spraying. Cytokines and chemokines not included in the table did not show any differential pattern according to any variable

*NS* not significant

^a^Ratio in the mean cytokine/chemokine levels in older women (> 25 years) compared to younger women (15–20 years)

^b^Ratio in the mean cytokine/chemokine levels in multigravidae compared to primigravidae

^c^Ratio in the mean cytokine/chemokine levels of women with a pregnancy during the dry season compared to women with a pregnancy during the rainy season

^d^Effect only manifested in primigravidae women in stratified analysis

^e^Effect only manifested in multigravidae women in stratified multivariable analysis

Depending on the season during pregnancy, the magnitude of IL-1β, IL-6, TNF and IL-8 responses at delivery varied significantly for both peripheral and cord blood cells following incubation with iRBC and uRBC. Lower levels of these pro-inflammatory cytokines and chemokines were significantly and consistently associated with the dry season (Table 3). In addition, cord IL-2 and IL-4 after stimulation with iRBC were also significantly lower in the dry season.

When examining the effect of malaria control tools on cytokine levels, CBMC from women sleeping under ITN produced higher levels of IL-10 than those not using ITN (Table 3). A weak interaction was noted between parity and ITN (p = 0.15); in the analyses stratified by parity the significant effect of ITN on IL-10 levels appeared to be attributable to MG women (Table 3).

Finally, maternal haematocrit was significantly associated with cord IL-8 in the adjusted analysis (ratio of mean IL-8 levels by haematocrit = − 2.46, 95% CI [− 4.28; − 0.63], p = 0.009 for iRBC, and − 2.83 [− 5.13; − 0.52], p = 0.018 for uRBC).

### Association of cytokines and chemokines with malaria and pregnancy outcomes

Lower production of IL-1β by PBMC following incubation with iRBC and uRBC was significantly associated with maternal peripheral infection (Table 4). Some interactions between peripheral infection and parity were noted for TNF (p = 0.054), IL-12 (p = 0.061), IL-2 (p = 0.003), IFN-γ (p = 0.025), IL-8 (p = 0.151), and IL-5 (p = 0.031). In the analysis stratified by parity, higher TNF and lower IL-12 secretion after iRBC and uRBC stimulations were significantly associated with maternal peripheral infection in PG women (Table 4). In contrast, lower IL-8 and higher IL-2 were associated with peripheral infection in MG women (Table 4). Such associations were not observed for CBMC stimulations (p > 0.05).

**Table 4:** Multivariable analysis of concentrations of cytokines and chemokines secreted by peripheral blood mononuclear cells in relation to *P. falciparum* infection and delivery outcomes

	Effect (95% CI)	p
Maternal peripheral *P. falciparum* infection^a^		
IL-1β		
iRBC	0.83 (0.72;0.97)	0.017
uRBC	0.86 (0.75;0.98)	0.026
TNF^c^		
iRBC	1.57 (1.01;2.45)	0.044
uRBC	1.70 (1.11;2.60)	0.015
IL-12^c^		
iRBC	0.38 (0.18;0.80)	0.015
uRBC	0.26 (0.08;0.79)	0.023
IL-8^d^		
iRBC	NS	
uRBC	0.71 (0.52;0.98)	0.035
IL-2^d^		
iRBC	NS	
uRBC	1.54 (1.03;2.28)	0.034
Placental *P. falciparum* infection^b^		
IL-6		
iRBC	0.89 (0.80;1.00)	0.049
uRBC	0.88 (0.79;0.98)	0.020
TNF		
iRBC	NS	
uRBC	0.82 (0.70;0.96)	0.015
IL-5^d^		
iRBC	1.80 (1.15;2.80)	0.009
uRBC	NS	
Placental inflammation		
TNF		
iRBC	1.35 (1.02;1.78)	0.034
uRBC	1.38 (1.06;1.79)	0.018
Cord *P. falciparum* infection		
IL-1β		
iRBC	3.58 (1.03;12.41)	0.045
uRBC	1.83 (1.01;3.32)	0.046

Adjusted for age, parity, indoor residual spraying, insecticide treated net, season, and neighbourhood. In some cases, the analyses were also adjusted for placental or peripheral infection, as indicated in the footnotes and in the text. The analyses were conducted first in all mothers and later stratified in primigravidae and multigravidae women separately, as indicated in the footnotes and in the text

*NS* not significant, *iRBC Plasmodium falciparum* infected red blood cells, *uRBC* uninfected red blood cells

^a^Also adjusted for placental infection

^b^Also adjusted for peripheral infection

^c^Effect only manifested in primigravidae women in stratified multivariable analysis

^d^Effect only manifested in multigravidae women in stratified multivariable analysis

When taking into account parasite density in infected women, only IL-5 production by CBMC correlated inversely with parasitaemia by microscopy (Spearman’s ρ = − 0.67, p = 0.023). In contrast, when parasites were quantified by qPCR, a consistent inverse correlation emerged for PBMC T_H_1 cytokines IFN-γ (ρ = − 0.34, p = 0.06), IL-2 (ρ = − 0.35, p = 0.070) and IL-12 (ρ = − 0.41, p = 0.025), and a direct correlation for PBMC TNF (ρ = 0.38, p = 0.042) and CBMC T_H_2 cytokines IL-4 (ρ = 0.40, p = 0.032) and IL-5 (ρ = 0.36, p = 0.060), but no association was shown for IL-1β, IL-6, IL-10 or any other cytokine. Geometric mean parasite densities by qPCR but not by microscopy were significantly higher in women with placental inflammation (10.38 parasites/µL, standard deviation 4.62) than without (0.46 parasites/µL, standard deviation 1.63, p = 0.034).

Placental infection was significantly associated with lower production of IL-6 and TNF by PBMC (Table 4). Higher IL-5 production after iRBC stimulation was significantly associated with placental infection in MG (p = 0.023, Table 4). Placental inflammation was significantly associated with higher TNF secretion by PBMC (Table 4), only significant for PG in the stratified analysis.

Cord blood infection was significantly associated with higher concentrations of IL-1β secreted by PBMC (Table 4). It was not possible to perform this multivariable analysis stratified by parity due to the low number of congenital infections (n = 8: 2 PG, 6 MG).

Finally, cytokine or chemokine concentrations were not significantly associated with maternal haematocrit, birth weight or incidence of clinical malaria in the children (p > 0.05).

## Discussion

The main finding of this study is that IL-6 and IL-1β were associated with a reduced risk of *P. falciparum* infection in pregnant women. Pro-inflammatory cytokines IL-6, IL-1β and TNF strongly correlated among themselves. However, higher production of IL-1β by PBMC was also significantly associated with congenital malaria after adjusting for potential confounding variables. In addition, excessive TNF, particularly in PG, was associated with peripheral infection and placental lesions. The apparently paradoxical association of IL-1β with both positive and negative outcomes could be reconciled if the effects on maternal periphery vs placental-fetal microenvironments may differ depending on concentration thresholds and locations. Thus, a given cytokine could be beneficial against parasites at a given concentration in a given tissue, while at certain other concentrations, and combined with other factors (e.g. placental inflammation) could be detrimental in other tissues, possibly favouring mother-to-child *P. falciparum* transmission.

Lower IL-1β secretion by PBMC was associated with peripheral infection at delivery and lower IL-6 secretion by PBMC was associated with placental infection during pregnancy, supporting the hypothesis that these pro-inflammatory cytokines may contribute to control maternal parasitaemia. However, these correlations did not translate into a clinical effect, which could be due to insufficient study power. Furthermore, in the parity-stratified analyses, some of these associations were only significant in MG mothers, suggesting that higher pro-inflammatory cytokines might lead to less malaria in a parity-dependent manner. Past studies showed that the ability to control malaria parasitaemia is predominantly age-dependent, suggesting naturally acquired immunity [10]. The finding that IL-1β and IL-6 increase in older pregnant women, and that this was *P. falciparum* antigen-specific in the multivariable analyses, further supports that these cytokines might be markers of acquired anti-malarial immunity in the mothers. However, there was also a clear and consistent effect of seasonality on the concentrations of secreted IL-1β, IL-6, TNF, and IL-8 from peripheral and fetal cells at delivery, that were higher when pregnancy occurred in the rainy season. This may indicate that these cytokines could also be markers of exposure to *P. falciparum*. Nevertheless, fluctuation of cytokines and chemokines might not be solely related to malaria, but to other factors affected by seasonality in Manhiça (e.g. respiratory infections, nutritional status). Unfortunately, phenotypic data from PBMC to check compositional variations according to season were not available.

An excessive production of pro-inflammatory cytokines may damage host tissues and it is believed that as part of the acquisition of immunity, the regulation by the anti-inflammatory cytokine IL-10 is key in preventing pathology. This is consistent with the finding that IL-1β, IL-6 and TNF were highly correlated among themselves and moderately correlated with IL-10, though TNF to a lesser extent. In fact, higher TNF production by PBMC, mostly in PG women, was associated with peripheral infection at delivery and placental inflammation, suggesting that in less immune pregnant women TNF production might be exacerbated during malaria and perhaps not well regulated by IL-10, becoming a marker of placental lesions. Other studies found TNF to be associated with placental malaria [46]. Thus, inflammation may result in increased levels of TNF, which in turn may cause more inflammation.

Increased IL-1β production by PBMC cultured with iRBC and uRBC was also associated with cord infection. In this cohort, congenital infection was significantly associated with placental inflammation and peripheral infection at delivery (Mayor et al., pers. comm.). In addition, higher peripheral parasite density by qPCR was associated with higher TNF and placental inflammation. Higher parasitaemias and lack of appropriate regulation by IL-10 may exacerbate IL-1β, IL-6 and TNF, leading to more placental inflammation and pathogenesis. Under those circumstances, it can be speculated that the integrity of placental tissue may become more compromised, being more likely that parasites may leak from the maternal side to cord blood.

Significantly less levels of IL-1β, IL-6, TNF and IL-10, but more IFN-γ secreted in response to iRBC, have been detected in grand MG (> 5 pregnancies) compared to women in their second to fourth pregnancy (44), giving insight into the modulation of PBMC function with increasing parity and on the balance between host protection and immunopathology in placental malaria. In contrast to some studies, increased IFN-γ did not correlate with protection against placental malaria [14–17]. However, an association between low levels of IL-12 and peripheral infection in PG was found, and IL-12 induces IFN-γ production. In addition, increased secretion of IL-5 by PBMC was significantly associated with placental infection in MG after parity stratification (consistent with [22, 23]), and this fits with the concept of protective T_H_1 *vs* non-protective T_H_2 for control of placental parasitaemia. Consistently, an inverse correlation between peripheral parasitaemia by qPCR and IL-12, IFN-γ and IL-2 (T_H_1), and a direct correlation between parasite density and IL-4 and IL-5 (T_H_2), were identified.

Regarding fetal responses, IL-10 production by CBMC was elevated in women sleeping under ITNs, particularly MG. The effect of ITN use on malaria incidence in mothers may influence IL-10 production in fetal cells, and this phenomenon may be modulated by parity-acquired immunity [47–49]. Also, CBMC from MG women secreted more IL-8 than CBMC from PG women, and lower maternal haematocrit was significantly associated with elevated production of IL-8 by CBMC. Although IL-8 was not assessed in placental tissue, the findings support that IL-8 is involved in the negative outcomes of malaria in pregnancy [19, 50, 51].

Some relevant cytokine or chemokine responses might not necessarily be *P. falciparum*-specific, as spontaneous release of pro-inflammatory cytokines was also found in control stimulations. In general, there was not a great difference between iRBC and uRBC stimulated samples. This suggests that in many cases we were measuring non-specific responses and/or that lysate stimulations are often not potent enough. Unfortunately, the study did not include a mitogen control in the stimulations due to limitation in cell numbers. In Manhiça, it is not feasible to have continuous production of fresh iRBC from in vitro parasite cultures to be used as antigens, which are more potent stimuli ex vivo. Nevertheless, non-specific cytokine responses are important and potentially relevant for disease outcomes, including malaria, as seen in this and other studies (Y. Song et al., pers. comm.), and should not be underestimated in the analyses as part of the overall action of innate and adaptive immunity.

No robust associations were found between cytokines or chemokines measured in maternal or cord blood and newborn or infant outcomes. Placental high TNF and low IFN-γ and IL-5 levels have been associated with low birth weight [46, 52]. Only newborns ≥ 2 kg were recruited as per trial criteria, which may account for the lack of associations in infant outcomes.

A limitation of this study is that only PBMC and CBMC but no placental blood cells were analysed, and there are immunological differences between peripheral and placental compartments, also affected by parity. As a result of this, placental qPCR and immune responses were not investigated due to the difficulty of obtaining fresh placental samples, although classical histology was performed. Thus, in this analysis, peripheral parasitaemia included submicroscopic infections and reflected diagnosis at delivery, while placental malaria included histological presence of pigment or past infections, and reflected diagnosis during pregnancy. In consequence, the protective association of IL-1β refers to the delivery while that of IL-6 includes the previous period of gestation. The restricted numbers of active placental malarias precluded from evaluating potential associations between presence of infections at delivery and cytokines. Future work should include flow cytometry characterization of cell populations producing the cytokines and chemokines. Finally, multiple associations were evaluated and chance in some of them cannot be excluded, thus findings must be taken with caution and confirmed in larger studies. Nevertheless, the internal consistency and biological plausibility of results generates confidence that data are robust.

## Conclusions

The most consistent finding of this study was the inverse association between increased levels of pro-inflammatory cytokines and maternal infection, and at the same time the direct association between IL-1β and cord infection, and between TNF, peripheral infection and placental inflammation in PG. Although no clear evidences for impact on child morbidity were found, findings highlight the complexity of immuno-pathological cytokine networks that might be acting in the placental and utero environments during malaria in pregnancy, with a potential trade-off between positive and negative effects on the mother and newborn susceptibility to *P. falciparum* infection, modulated by parity.

## Additional files

**Additional file 1.** Combination between the presence of peripheral, placental and cord malaria infections as well as with placental inflammation.

**Additional file 2.** Correlations among cytokines and chemokines secreted by cord blood mononuclear cells in culture supernatants.

**Additional file 3.** Correlations among mRNA cytokines produced by peripheral blood mononuclear cells.

**Additional file 4.** Correlations between cytokines and chemokines produced by peripheral blood mononuclear cells (PBMC) and cord blood mononuclear cells (CBMC) in culture supernatants.

**Additional file 5.** Correlations between cytokines and chemokines produced by blood mononuclear cells.

## Abbreviations

IFN: interferon
IL: interleukin
TNF: tumor necrosis factor
T_H_: T helper lymphocyte
Treg: T regulatory
mRNA: messenger RNA
cDNA: complementary DNA
PBMC: peripheral blood mononuclear cells
CBMC: cord blood mononuclear cells
iRBC: infected red blood cells
uRBC: uninfected red blood cells
qPCR: real-time quantitative PCR
RT-qPCR: reverse transcriptase qPCR
CISM: Centro de Investigação em Saúde de Manhiça
MDH: Manhiça District Hospital
MHP: Maragra Health Post
HIV: human immunodeficiency virus
QIMR: Queensland Institute of Medical Research
PG: primigravidae
MG: multigravidae
ITN: insecticide-treated net
IRS: indoor residual spraying
