# Supplementary material for: High production of pro-inflammatory cytokines by maternal blood mononuclear cells is associated with reduced maternal malaria but increased cord blood infection
Source: Malar J. 2018 May 10;17:177. doi: 10.1186/s12936-018-2317-2 (PMC5944101; doi:10.1186/s12936-018-2317-2)
Supplement: Supplementary file 1 — Additional file 1. Combination between the presence of peripheral, placental and cord malaria infections as well as with placental inflammation. [file 12936_2018_2317_MOESM1_ESM.docx]

**Additional file 1**. **Combination between the presence of peripheral, placental and cord malaria infections as well as with placental inflammation.**

|  |  | **Placental infection** | | |  |  |  |
| --- | --- | --- | --- | --- | --- | --- | --- |
| **Peripheral infection** | | Negative | Positive | Total |  |  |  |
|  | Negative | 120 | 21 | 141 |  |  |  |
|  | Positive | 17 | 14 | 31 |  |  |  |
|  | Total | 137 | 35 | 172 |  | Fisher's exact test = | 0.000 |
|  |  |  |  |  |  |  |  |
|  |  | **Cord infection** | |  |  |  |  |
| **Peripheral infection** | | Negative | Positive | Total |  |  |  |
|  | Negative | 135 | 6 | 141 |  |  |  |
|  | Positive | 31 | 0 | 31 |  |  |  |
|  | Total | 166 | 6 | 172 |  | Fisher's exact test = | 0.593 |
|  |  |  |  |  |  |  |  |
|  |  | **Placental inflammation** | | | |  |  |
| **Peripheral infection** | | Negative | Positive | Total |  |  |  |
|  | Negative | 136 | 5 | 141 |  |  |  |
|  | Positive | 27 | 4 | 31 |  |  |  |
|  | Total | 163 | 9 | 172 |  | Fisher's exact test = | 0.057 |
|  |  |  |  |  |  |  |  |
|  |  | **Cord infection** | |  |  |  |  |
| **Placental infection** | | Negative | Positive | Total |  |  |  |
|  | Negative | 134 | 3 | 137 |  |  |  |
|  | Positive | 32 | 3 | 35 |  |  |  |
|  | Total | 166 | 6 | 172 |  | Fisher's exact test = | 0.1 |
|  |  |  |  |  |  |  |  |
|  |  | **Placental inflammation** | | | |  |  |
| **Placental infection** | | Negative | Positive | Total |  |  |  |
|  | Negative | 131 | 6 | 137 |  |  |  |
|  | Positive | 32 | 3 | 35 |  |  |  |
|  | Total | 163 | 9 | 172 |  | Fisher's exact test = | 0.39 |
