# Supplementary material for: High production of pro-inflammatory cytokines by maternal blood mononuclear cells is associated with reduced maternal malaria but increased cord blood infection
Source: Malar J. 2018 May 10;17:177. doi: 10.1186/s12936-018-2317-2 (PMC5944101; doi:10.1186/s12936-018-2317-2)

**Additional file 3**. **Correlations among mRNA cytokines produced by peripheral blood mononuclear cells.** Stimulation with a lysate of *P. falciparum* infected erythrocytes (A) and a lysate of uninfected erythrocytes (B).

**A)** Stimulation with a lysate of *P. falciparum* infected erythrocytes


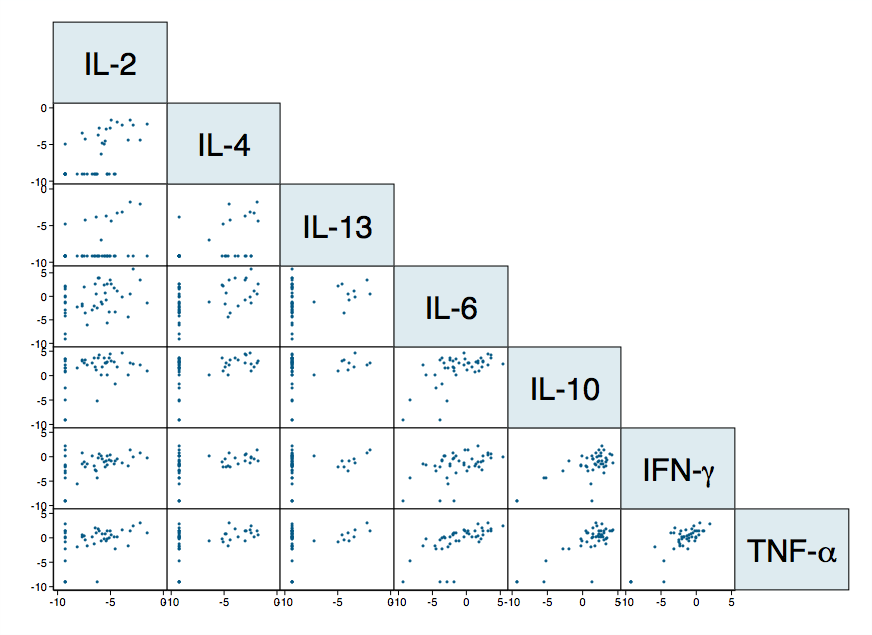


**B)** Stimulation with a lysate of uninfected erythrocytes


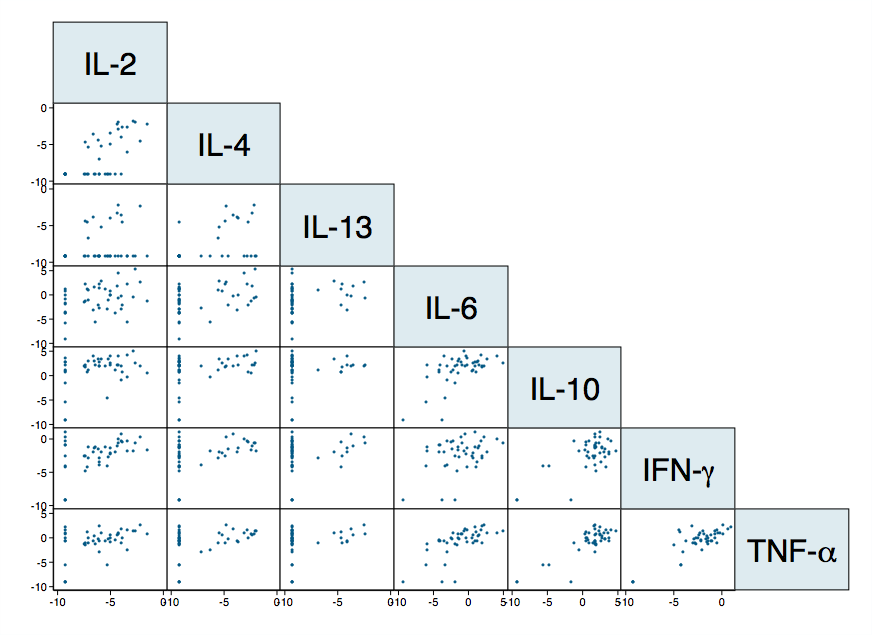

Supplement: Supplementary file 3 — Additional file 3. Correlations among mRNA cytokines produced by peripheral blood mononuclear cells. [file 12936_2018_2317_MOESM3_ESM.docx]
