# Supplementary material for: High production of pro-inflammatory cytokines by maternal blood mononuclear cells is associated with reduced maternal malaria but increased cord blood infection
Source: Malar J. 2018 May 10;17:177. doi: 10.1186/s12936-018-2317-2 (PMC5944101; doi:10.1186/s12936-018-2317-2)

**Additional file 4**. **Correlations between cytokines and chemokines produced by peripheral blood mononuclear cells (PBMC) and cord blood mononuclear cells (CBMC) in culture supernatants.** Stimulation with a lysate of *P. falciparum* infected erythrocytes (A) and a lysate of uninfected erythrocytes (B), showing the rho coefficients and p values (Bonferroni corrected).

**A)** Stimulation with a lysate of *P. falciparum* infected erythrocytes


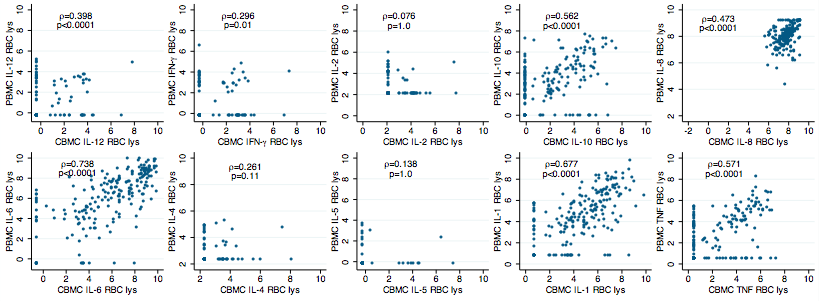


**B)** Stimulation with a lysate of uninfected erythrocytes


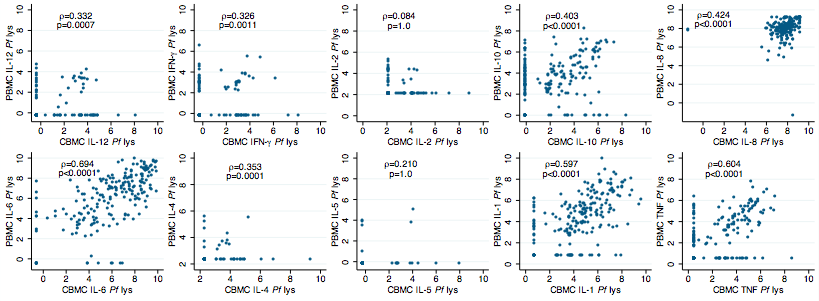

Supplement: Supplementary file 4 — Additional file 4. Correlations between cytokines and chemokines produced by peripheral blood mononuclear cells (PBMC) and cord blood mononuclear cells (CBMC) in culture supernatants. [file 12936_2018_2317_MOESM4_ESM.docx]
