# Supplementary material for: High production of pro-inflammatory cytokines by maternal blood mononuclear cells is associated with reduced maternal malaria but increased cord blood infection
Source: Malar J. 2018 May 10;17:177. doi: 10.1186/s12936-018-2317-2 (PMC5944101; doi:10.1186/s12936-018-2317-2)

**Additional file 5**. **Correlations between cytokines and chemokines produced by blood mononuclear cells.** Stimulation with a lysate of *P. falciparum* infected erythrocytes (X axis) and a lysate of uninfected erythrocytes (Y axis), in culture supernatants (A) and as mRNA in cells (B), showing the rho coefficients and p values (Bonferroni corrected).

**A) Culture supernatants**


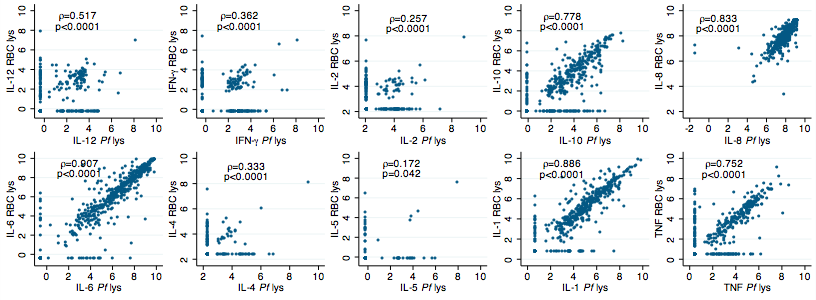


**B) mRNA**


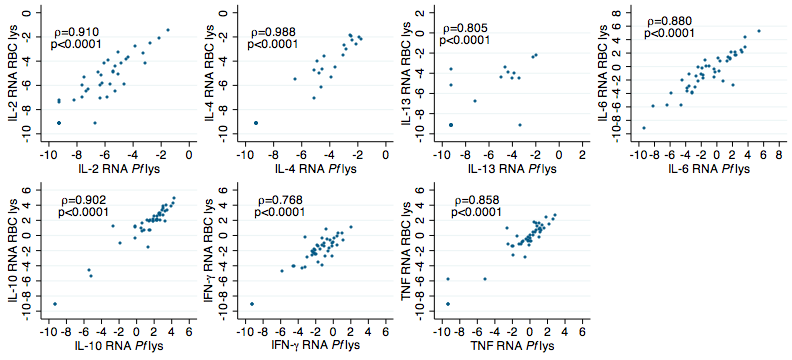

Supplement: Supplementary file 5 — Additional file 5. Correlations between cytokines and chemokines produced by blood mononuclear cells. [file 12936_2018_2317_MOESM5_ESM.docx]
